# Defense behavior of two closely related but geographically distant host species against cuckoo parasitism: A next test for the parallel coevolution

**DOI:** 10.1002/ece3.10175

**Published:** 2023-06-09

**Authors:** Alfréd Trnka, Laikun Ma, Hanlin Yan, Longwu Wang, Wei Liang

**Affiliations:** ^1^ Department of Biology University of Trnava Trnava Slovakia; ^2^ School of Life Sciences Hebei University Baoding China; ^3^ Department of Biology and Food Science Hebei Normal University for Nationalities Chengde China; ^4^ Ministry of Education Key Laboratory for Ecology of Tropical Islands, Key Laboratory of Tropical Animal and Plant Ecology of Hainan Province, College of Life Sciences Hainan Normal University Haikou China; ^5^ School of Life Sciences Guizhou Normal University Guiyang China

**Keywords:** 3D model, avian brood parasitism, coevolution, dummy experiment, egg rejection

## Abstract

Interactions between avian brood parasites, such as common cuckoos (*Cuculus canorus*), and their hosts are one of the best‐studied examples of the coevolutionary arms race. Different stages of this arms race can be seen in different races of common cuckoos and their hosts across their range. However, little is known whether selected populations of two closely related but geographically distant species with probably different coevolutionary histories with the common cuckoo are also at different stages of the arms race. In this study, we tested this prediction experimentally using the same non‐mimetic model eggs and three‐dimensional (3D) printed models of the gray adult common cuckoo (*Cuculus canorus*). We examined egg recognition and egg rejection and aggression against the common cuckoo in the great reed warbler (*Acrocephalus arundinaceus*) and Oriental reed warbler (*Acrocephalus orientalis*), in Slovakia of Europe and northeast China of Asia. The results showed that the great reed warbler exhibited stronger responses to experimental model eggs and 3D models of the common cuckoo than the Oriental reed warbler. We conclude that both the great reed warbler and Oriental reed warbler have well‐developed antiparasitic behaviors against common cuckoos in the studied populations, but with different levels of defense intensity, which may be due to local differences in parasitic pressure and the risk of parasitism. This provides an opportunity to study coevolutionary processes between the brood parasite and its hosts together in both species at large geographical scales.

## Graphical abstract

We experimentally use the same eggs and models to compare egg recognition and egg rejection and aggression against cuckoos in its two hosts, the great reed warbler (*Acrocephalus arundinaceus*) and Oriental reed warbler (*Acrocephalus orientalis*), in Europe and China. The great reed warbler exhibited stronger responses to experimental model eggs and 3D models of the common cuckoo than the Oriental reed warbler. Both the great reed warbler and Oriental reed warbler have well‐developed antiparasitic behaviors against cuckoos but with different levels of defense intensity, which may be due to local differences in parasitic pressure and the risk of parasitism.

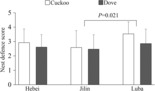

## 1 INTRODUCTION

Coevolution, a reciprocal evolutionary process between interacting species driven by natural selection, plays a key role in shaping the biodiversity on our planet (Thompson, 1994). Therefore, it has long been of interest to scientists. However, because different factors, such as the environment and especially predation or parasitism pressures, can have different effects on ways in which species exposed to them evolve, there can be various types of evolution with often only subtle differences. Unlike convergent evolution, for example, when unrelated or only distantly related species evolved the same traits in similar environment, parallel (co)evolution predicts a correlation between phenotypes of closely related species and their environments (Schwartz & Hendry, 2007), although there may also be a continuum between these two types of evolution (Arendt & Reznick, 2008). Hence, according to above‐mentioned prediction, two closely related species should show similar phenotypic adaptation to similar ecological environments, including predators and parasites, regardless of their evolutionary history, and geographical distribution.

One of the well‐known model systems for the study of coevolution is avian interspecific brood parasitism in which individuals of one species (the brood parasite) exploit individuals of the other species (the host) to raise their offspring. This is because of high fitness costs imposed by brood parasitism on hosts selected for a variety of highly sophisticated adaptations and counter‐adaptations between interacting species, resembling an arms race (Davies, 2011; Feeney et al., 2014; Soler, 2017). The most widespread defensive mechanisms evolved by hosts against brood parasitism are recognition of and aggression toward adult parasites near the host nest and recognition and rejection of parasite eggs (Davies, 2000; Feeney et al., 2012). However, while the ability to recognize a brood parasite as a nest threat appears to be a learned behavior (Abernathy et al., 2021; Langmore et al., 2012), egg‐recognition ability is likely to be genetically based, although the learning process is also necessary for fine‐tuning of recognition (Martínez et al., 2020; Martín‐Gálvez et al., 2006; Rothstein, 1990; Soler et al., 1999; but see Procházka et al., 2014). Thus, unlike the evolution of egg rejection, which can take at least 30–100 years—the higher the rate and cost of parasitism, the faster the evolution (Abernathy et al., 2021), the recognition of adult brood parasite, if socially transmitted (e.g., Davies & Welbergen, 2009; Feeney & Langmore, 2013) can spread much more rapidly throughout a host population. Comparison of the antiparasite defenses of two closely related host species in distant areas can therefore help us better understand coevolutionary interactions between hosts and their parasites.

An initial step in the parallel coevolution between two closely related hosts was made by Moskát et al. (2012), who investigated egg rejection and egg mimicry in the common cuckoo (*Cuculus canorus*) and its two hosts, the great reed warbler (*Acrocephalus arundinaceus*) and the Oriental reed warbler (*Acrocephalus orientalis*) in Hungary and Japan. Both species showed moderately evolved antiparasitic defenses at the study sites. Nevertheless, despite similar egg rejection rates in the two host species, cuckoo eggs found in Hungary showed better mimicry to host eggs than cuckoo eggs found in Japan, which suggests that coevolutionary mechanisms may have different speeds and the common cuckoo and its hosts may be at different stages in arms race in the two distant areas. However, although egg recognition and egg rejection are important indicators of host adaptation to brood parasitism (Davies & Brooke, 1989a; Ma & Liang, 2021), efficiency of other lines of defense against parasitism in the studied species need to be compared to generalize obtained findings.

In this study, we, therefore, focused specifically, in addition to egg rejection behavior, on comparing the recognition ability and aggression against adult common cuckoos in the great reed warbler and Oriental reed warbler at selected study sites in Slovakia (central Europe) and northeast China, which has not been studied to date. The great reed warbler is one of the largest species in the family Acrocephalidae breeding throughout mainland Europe and the west Palearctic and wintering in Sub‐Saharan Africa. It is a facultatively polygynous species, with rates of social polygyny ranging from 8% to 56% (Catchpole et al., 1985; Hasselquist, 1998; Koleček et al., 2015; Leisler & Wink, 2000). Only the female builds the nest and incubates the clutch, however, both females and males defend the nest (Požgayová et al., 2009; Trnka & Grim, 2013). In Slovakia, the great reed warbler is a common host of the common cuckoo, with an annual parasitism rate between 24% and 48.5% in the study area (Trnka, Požgayová, et al., 2012; Trnka et al., 2013; Trnka & Grim, 2014).

The Oriental reed warbler, formerly considered a subspecies of the great reed warbler (Dyrcz & Nagata, 2002), is slightly smaller and slenderer than the great reed warbler. Its breeding range covers southern Siberia, Mongolia, northeastern China, Korea, and Japan. The species winters in South Asia, North Australia, and Philippines. The rate of social polygyny varies between 14% and 57% (Choi et al., 2010; Urano, 1995). Similar to the great reed warbler, only the female builds the nest and incubates the eggs, but males participate in nest defense. The nests of Oriental reed warbler are frequently parasitized across China with different parasitism rates (14.7%–65.5%; Li, Ruan, et al., 2016; Li, Zhang, et al., 2016; Li et al., 2020; Liang et al., 2014; Yang et al., 2014). Both species show the ability to recognize and reject common cuckoo eggs (Li, Zhang, et al., 2016; Ma & Liang, 2021; Trnka, Požgayová, et al., 2012; Trnka & Grim, 2014; Yang et al., 2016, 2017) and behave aggressively toward adult common cuckoos near their nests (Li et al., 2015, Li, Zhang, et al., 2016; Ma, Yang, & Liang, 2018; Ma, Yang, Liu, et al., 2018; Trnka, Prokop, et al., 2012, Trnka et al., 2013; Trnka & Prokop, 2012) at varying levels of intensity.

Considering that great reed warblers and Oriental reed warblers occupy similar reed bed habitats and are regularly parasitized in the study sites in Slovakia and China, we predicted that egg rejection behavior and aggression against cuckoos are also similar in these two host species, and hence, they achieve a similar level of coevolutionary adaptations regardless of their origin and geographic distance. We tested this prediction experimentally using non‐mimetic model eggs and three‐dimensional (3D) printed models of adult common cuckoos and Oriental turtle doves (*Streptopelia orientalis*) as stimuli to standardize experimental conditions in both study areas. Artificial eggs and dummies of brood parasites, including 3D models, are used commonly and successfully also in other host egg rejection and host aggression studies (Chen et al., 2022; Hauber et al., 2015; Igic et al., 2015; Samaš et al., 2021; Tryjanowski et al., 2018, but see Yang et al., 2020).

## 2 MATERIALS AND METHODS

### 2.1 Study site and general field procedures

The study was carried out in 2022 at one site in Slovakia, a fish pond system near Štúrovo, southwestern Slovakia (47°51′ N, 18°36′ E, 115 m above sea level, thereafter Luba), and two sites in northeast China, Baiyangdian Lake, Hebei (38°43′–39°10′ N, 115°38′–116°19′ E, 10 m above sea level, thereafter Hebei) and Sifangtuozi Farm, Jilin (46°12′ N, 123°84′ E, 129 m above sea level, thereafter Jilin). The two sites in China are about 1300 km apart.

The Luba study site is located in a warm, temperate climate zone, with an annual average air temperature of 10.4°C (min–max: −35.0–40.3°C) and annual average precipitation of 556 mm. The great reed warbler breeds here in narrow, approximately 5–10 m wide, strips of the common reed (*Phragmites australis*) that border the ponds. In the study year, the great reed warbler population comprised 40–50 breeding pairs and the parasitism rate by the common cuckoo reached 35.7% (*N* = 42).

The study site Hebei represents the largest freshwater shallow lake in the north China Plain, located in a warm, temperate, and semi‐arid continental monsoon climate zone. The annual average air temperature here is 12.2°C, with an extreme maximum temperature of 40.7°C and an extreme minimum temperature of −26.7°C. The annual average precipitation and evaporation are 529.7 and 993.0 mm, respectively (He et al., 2022). The size of the breeding population of Oriental reed warblers is estimated at 120–140 pairs, and the estimated rate of cuckoo parasitism is 19.8% (*N* = 28/141). The birds breed here in reed wetlands.

The site Jilin is located in a semi‐arid temperate continental monsoon climate zone, with an annual average temperature of 4.4°C and average annual precipitation of 384–521 mm (Zhu et al., 2022). Estimated average size of breeding population is 80–120 pairs and the rate of cuckoo parasitism reaches 30.9% (*N* = 38/123). The habitats included reed wetlands, open waterbodies, and cultivated lands, and most Oriental reed warblers nested in reed wetlands.

To locate great reed warbler and Oriental reed warbler nests, we systematically searched the reed beds at 3–5 days intervals from May to July (Luba) and from June to August (Hebei and Jilin). Most nests were found during building or egg‐laying. Once a nest was found, we checked it at 2–3 days intervals to determine the day of clutch completion, the final clutch size and to detect the presence of a parasitic egg. We determined the current social mating status of each female and her mate based on their captures at the nests during the nest‐building or egg‐laying stages (Luba), or we tested only the first nest in each male's territory that could be regarded as the nest of a currently monogamous female (Hebei and Jilin). Note that monogamous females receive much more male nest defense assistance than females of polygynous males, which might influence their nest defense behavior (Požgayová et al., 2013; Trnka & Prokop, 2010). Therefore, all experiments we conducted on currently monogamous nests only.

### 2.2 Egg rejection experiment

Egg rejection behaviors of great reed warblers and Oriental reed warblers were investigated on a subset of randomly selected previously unparasitized nests. Nests were parasitized experimentally by replacing one of the host's eggs with one non‐mimetic blue model egg during egg laying. Blue model eggs were produced using a highly plastic synthetic soft clay (3.64 ± 0.03 g in egg mass, 22.75 ± 0.38 mm × 14.33 ± 0.19 in egg size, *n* = 23), similar to the size of local cuckoo eggs (3.05 ± 0.09 g in egg mass, 21.80 ± 0.55 mm × 16.22 ± 0.40 in egg size, *n* = 27; see Ma & Liang, 2021). We selected blue color for the experimental eggs because this is a conspicuous color unlike the egg color of the tested hosts. In addition, blue model eggs better mimic the ultraviolet part of the spectrum of host eggs and appear more likely to the green‐blue background of the natural eggs of *Acrocephalus* warblers than, for example, brown spotted eggs (Li et al., 2020). We did not use a control (simulated debris) to test whether the observed egg rejection rate was a result of the nest sanitation behavior of hosts or a defense against brood parasitism since hosts including the great reed warbler and Oriental reed warbler generally consider artificial egg‐shaped objects to be eggs (see also Honza & Moskát, 2008; Li et al., 2020; Ma & Liang, 2021; Moskát et al., 2002). The experimental egg was left in the nest for 6 days. The egg was scored as accepted if it remained undamaged in the nest and the nest was not abandoned by the 6th day and rejected if it disappeared from the nest but the clutch was incubated. Predated or destroyed nests within these 6 days were excluded from subsequent analyses.

### 2.3 Nest defense experiment

Nest defense by hosts was tested with 3D printed models of the gray common cuckoo and the Oriental turtle dove as a control (two models from each bird species). The model size of the two species is similar to real birds about 28 and 30 cm, respectively. And the tail of the cuckoo model is upturned to simulate the resting position in nature. We applied sequential randomized presentation of models at host nests. The time lag between the two presentations was at least 90 min to prevent habituation or carry‐over aggression of the tested birds. To minimize the risk of nest desertion caused by disturbing a female during egg laying, we conducted all experiments 1–2 days after clutch completion. The models were placed 0.5 m from the focal nest, at the height of the nest rim, with the head facing the nest. Observations were made by the same observer from a shelter placed 4–5 m away from the nest (Luba), and responses of hosts were video‐recorded with a micro camera (Uniscom‐T71, 70 × 26 × 12 mm; Mymahdi Technology Co., Ltd.) above the nest (Zhao et al., 2022) and subsequently analyzed (Hebei and Jilin).

The experiment started when the nest owner(s) appeared near the nest and spotted the model, and lasted for 5 min. If no parent was observed near the nest for 15 min since the model was exposed, the experiment was stopped and excluded from the analyses. Host behavior was quantified on the following scale: (1) no response, the bird silently watched the model from a safe place; (2) alert, the bird approached the nest giving short alert calls; (3) mobbing, the bird jumped closely around the model or flew over it, giving alarm and distress calls; (4) attack, the bird physically attacked the model, persistently uttering distress calls. As both the great reed warbler and Oriental reed warbler are sexually monomorphic and the color rings on the legs of birds are only difficult to identify due to their fast movements in dense reeds, we did not distinguish different individuals by sex during experiments. We used the maximum reaction of parents in the analysis. Both above‐mentioned procedures for quantifying egg‐rejection behavior and nest defense behavior were successfully used in the two study species also in our previous studies (e.g., Li et al., 2015; Ma, Yang, & Liang, 2018; Ma & Liang, 2021; Trnka & Grim, 2014; Trnka & Požgayová, 2017). In total, we performed the egg rejection experiments on 56 nests (27 nests in Luba and 29 nests in Hebei and Jilin) and nest defense experiments on 58 nests of different breeding pairs (28 nests in Luba and 30 in Hebei and Jilin).

### 2.4 Statistical analyses

Cumulative link mixed models (CLMMs, clmm in R package ordinal) with logit‐link function were used to predict the response (1 = no response, 2 = alert, 3 = mobbing, or 4 = attack) toward the dummy (cuckoo or dove model) in different study areas (Luba, Hebei, Jilin). And we used two‐tailed likelihood ratio tests to obtain *p* values. In the model, response scores were the dependent variable, whereas study site, clutch size, type of model, and the interactions between the study site and type of model were treated as fixed independent terms, and individuals distinguishing birds' nests were random independent terms. When we found significant differences in treatments or populations, we conducted new CLMMs for each treatment or each study site separately.

Fisher's exact test or Chi‐square test was used for comparison between different probabilities. All tests were two‐tailed, with a significance level of *p* < .05, and data are presented in the form of mean ± standard deviation (Mean ± SD).

## 3 RESULTS

### 3.1 Egg rejection behavior

**FIGURE 1:**
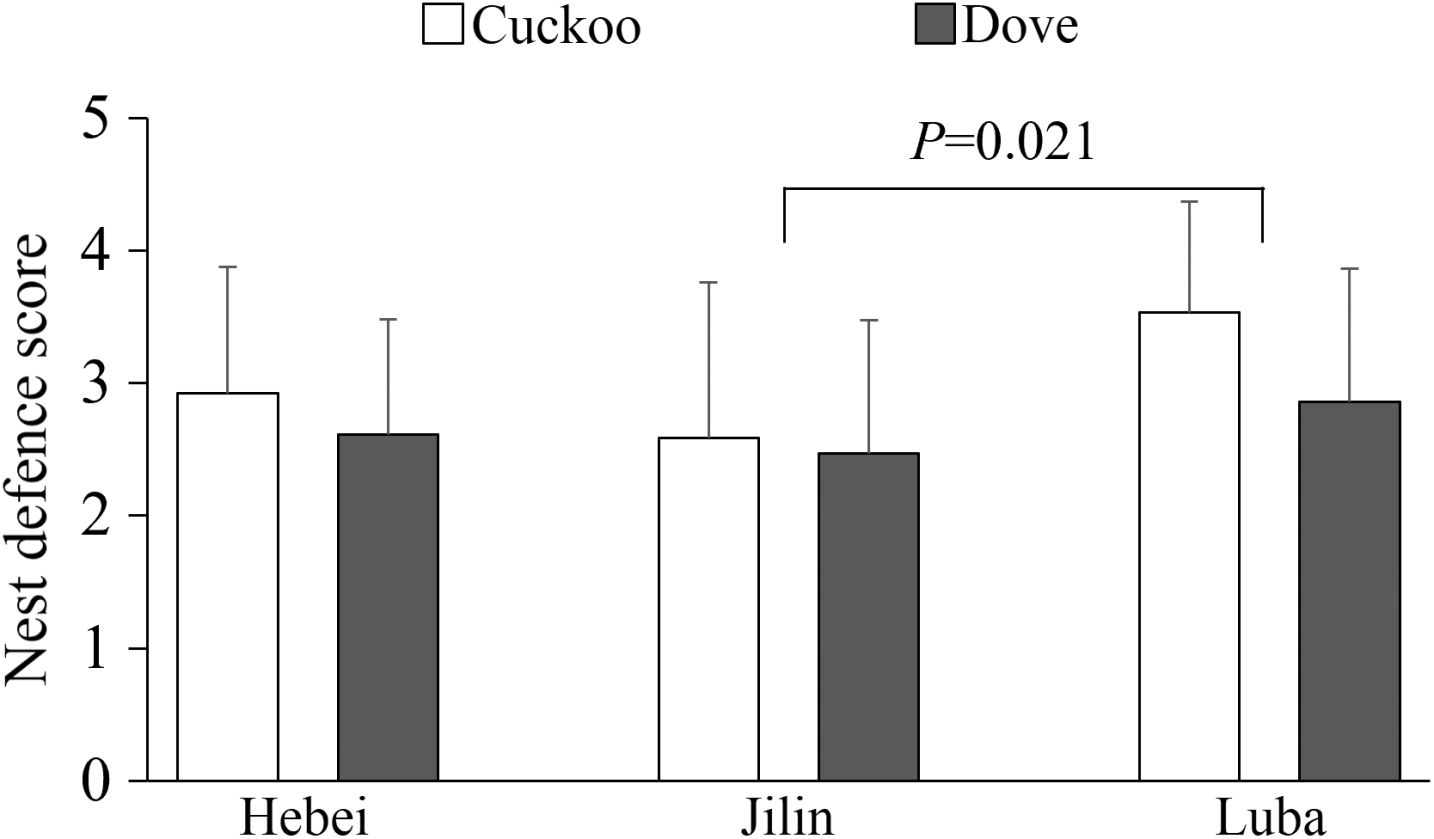
Egg rejection rates of cuckoo hosts among different study areas.

Out of 56 experimentally parasitized great reed warbler and Oriental reed warbler nests hosts rejected the blue model egg in 44 (78.6%) nests, most frequently by ejection (35 cases). There were significant differences in egg rejections among the three study sites (Fisher's exact test: *p* = .026), with egg rejection rate in Luba (92.6%) being higher than in Hebei and Jilin (63.6% and 66.6%, respectively; Fisher's exact test: *P*_1_ = .042, *P*_2_ = .039; Figure 1). Since there was no significant difference in egg rejections between Hebei and Jilin (Fisher's exact test: *p* = 1.000), data from the two sites were merged for further comparison with Luba. The result then indicated that the great reed warbler shows a much stronger egg rejection than the Oriental reed warbler (92.6% and 65.5%, respectively; Chi‐square test: χ^2^ = 6.788, *df* = 1, *p* = .009).

### 3.2 Nest defense behavior

**FIGURE 2:**
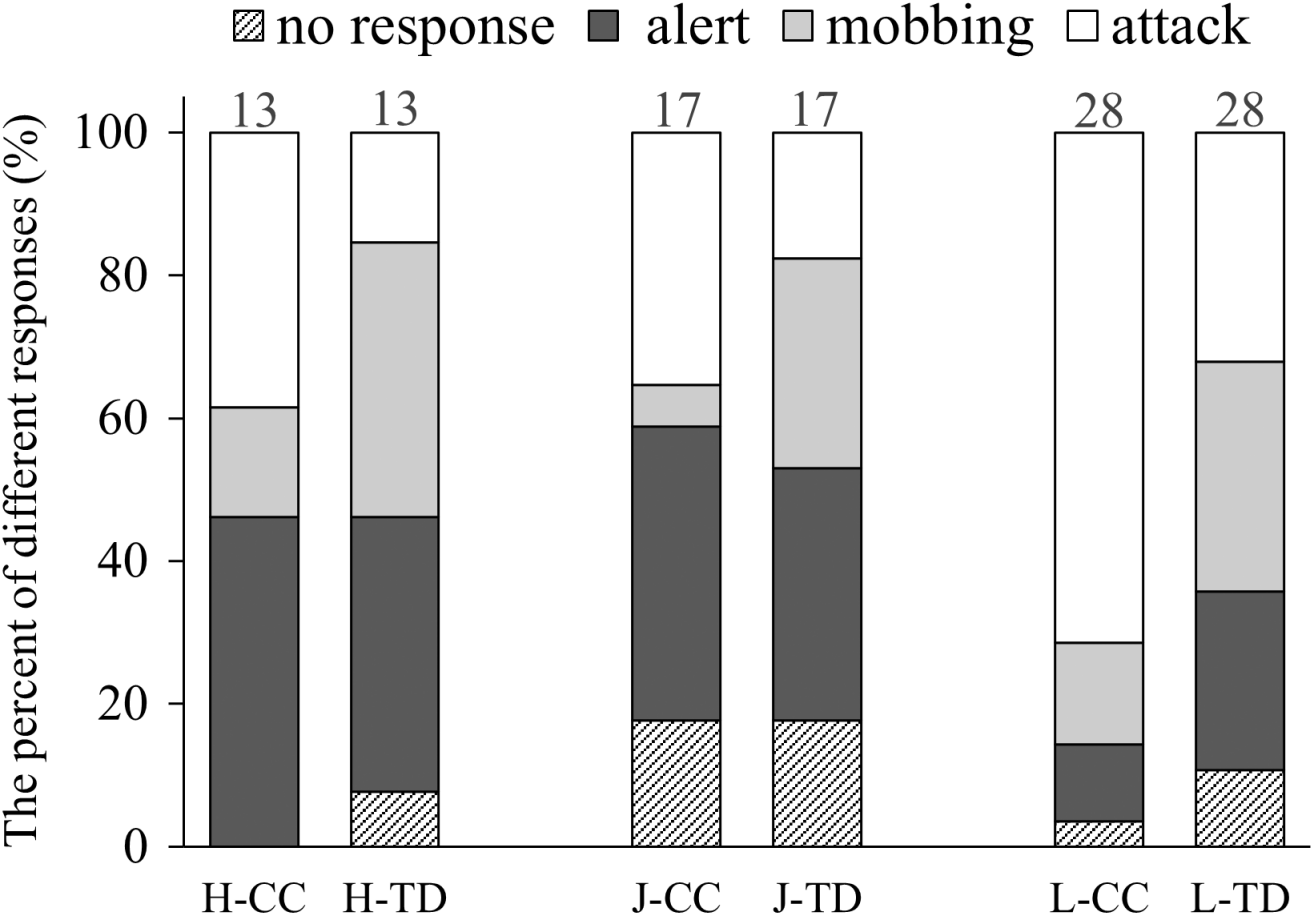
The percent of different responses to two types of 3D models among three study sites. H‐, J‐ and L‐ refer to the study site of Hebei, Jilin, and Luba, respectively. CC and TD refer to the common cuckoo and turtle dove, respectively. The number on the bar refers to the sample size.

General aggression of great reed warblers and Oriental reed warblers directed toward at least one of the two models (either common cuckoo or turtle dove) was apparent in 45 of 58 (77.6%) tested nests, direct body attacks were observed in 33 of 58 (56.9%) nests. CLMMs showed that study site ($x_{2}^{2}$ = 7.462, *p* = .024) and type of model ($x_{1}^{2}$ = 8.074, *p* = .004) were the two factors that could predict the response toward the model in different study areas (Table 1, Figure 2).

**TABLE 1:** Cumulative link mixed models (CLMMs) were used to predict the response toward the 3D models in different study areas.

	$x^{2}$	*Df*	*p*
Study site	7.462	2	.024*
Clutch size	0.058	1	.811
Type of model	8.074	1	.004*
Study site × Type of model	4.368	2	.113

* *p* < .05.

There was no difference between Hebei and Jilin in nest defense scores to two types of models (study site: $x_{1}^{2}$ = 0.010, *p* = .921; type of model: $x_{1}^{2}$ = 0.448, *p* = .503), then data of the two sites were merged for further comparison with Luba, and the results indicated that the interaction between species and type of model significantly affected the responses of birds ($x_{1}^{2}$ = 4.156, *p* = .041; Table 2). More specifically, great reed warblers physically attacked the cuckoo model in 20 of 28 (71.4%) nests and the dove model in 9 of 28 (32.1%) nests, whereas Oriental reed warblers directly attacked the model of the common cuckoo only in 11 of 30 (36.7%) nests and the model of the dove in 5 of 30 (16.7%) nests (Figure 2). However, the response intensity of great reed warbler to cuckoo model was significantly higher than that of dove ($x_{1}^{2}$ = 28.372, *p* < .001), whereas there was no significant difference in response intensity between cuckoo and turtle dove models for Oriental reed warbler ($x_{1}^{2}$ = 0.446, *p* = .504). In addition, the response intensity of great reed warbler to cuckoo model was significantly higher than that of Oriental reed warbler ($x_{1}^{2}$ = 10.245, *p* = .001), while the responses of the two species to the dove were not different ($x_{1}^{2}$ = 1.439, *p* = .230).

**TABLE 2:** Cumulative link mixed models (CLMMs) were used to predict the response toward 3D models by different host species (GRW vs. ORW).

	$x^{2}$	*Df*	*p*
Clutch size	0.324	1	.569
Type of model	8.131	1	.004**
Species	7.108	1	.008**
Species × Type of model	4.156	1	.041*

* *p* < .05

** *p* < .01.

## 4 DISCUSSION

Contrary to our prediction that egg rejection and aggression against common cuckoos are similar in the two closely related host species, the great reed warbler exhibited stronger responses to experimental model eggs and 3D models of the cuckoo than the Oriental reed warbler in our study sites in Slovakia and China. This also contrasts with the study of Moskát et al. (2012) that, despite differences in cuckoo egg mimicry, has reported similar rejection rates in the two *Acrocephalus* warbler populations in Hungary and Japan. However, in the previous study, the authors investigated the rejection of real cuckoo eggs in naturally parasitized host nests, whereas we used artificial non‐mimetic (blue) eggs in our egg rejection experiments, which could have skewed the results. Given that the degree of egg mimicry is an important factor in host recognition (Davies & Brooke, 1988, Yang et al., 2019), egg rejection rates of blue model eggs can be overestimated in the study species. Model eggs had not to be rejected in a predictable way also because of their artificial color and material from which they were made (Antonov et al., 2009; Hanley et al., 2017; Li et al., 2020; Roncalli et al., 2017). However, the chance that our experimental eggs avoided rejection due to difficulty in removing them is unlikely as evident from videos (see, e.g., Ma & Liang, 2021). Non‐mimetic model eggs, on the other hand, may have been relatively more tolerated in nests with lower uniformity of host egg appearance within a clutch (Davies & Brooke, 1989b; Moskát, Avilés et al., 2008), which we did not measure. Anyway, significant differences in rejection rates between great reed warblers and Oriental reed warbles in Slovakia and China (this study) and high variations in the rejection of blue model eggs among various geographic populations of the Oriental reed warbler in China (Li, Zhang, et al., 2016; Li et al., 2020; Ma & Liang, 2021; Wang et al., 2021, this study) indicate the validity of our results. This was also confirmed by our previous observations of rejection rates of natural cuckoo eggs in our study populations (65.7% in Slovakia vs. 4.3% in China, Trnka, Požgayová, et al., 2012; Wang et al., 2021). Thus, we believe that relative differences in the rejection of experimental blue eggs between the two studied species reflect real differences in the rejection rate of natural cuckoo eggs in our study areas.

Both species also showed different nest defense behavior against adult cuckoos. Great reed warblers responded more aggressively to the 3D cuckoo model than Oriental reed warblers, but both clearly discriminated between the cuckoo and the control dove suggesting fine‐tuned recognition ability of adult common cuckoos in these hosts. Obtained results correspond with the data presented by Moskát et al. (2012), but birds tested at our study sites exhibited much lower levels of aggression than those tested at the other localities in Europe and China (Bártol et al., 2002; Li et al., 2015; Ma, Yang, & Liang, 2018; Požgayová et al., 2013; but see Dyrcz & Hałupka, 2006). In addition to the different levels of parasitic pressure at the individual study sites, a different type of cuckoo model used in our study could have caused observed differences in host aggression. While in the previous studies, the authors used mainly taxidermic (stuffed) models of the common cuckoo, we used 3D printed models as a stimulus to observe host behavioral responses to a brood parasite. However, a recent study by Chen et al. (2022) showed that Oriental reed warblers respond similarly to taxidermy specimens and 3D‐printed models of the common cuckoo and turtle dove, which disproves this assumption.

Differences in egg rejection rates and aggression against adult common cuckoos between great reed warblers and Oriental reed warblers in Slovakia and China can also be partly explained by different stages of their coevolution with cuckoos, as was also found in Hungarian and Japanese populations of these two species (Moskát et al., 2012). However, despite we do not know local levels of cuckoo counter‐adaptation to hosts in our study sites, particularly the level of egg mimicry, Ma et al. (2022) and Li, Zhang, et al. (2016) demonstrated that common cuckoos in northern and eastern China have developed eggs with a high degree of mimicry to Oriental reed warbler eggs, even higher than that estimated by Moskát et al. (2012) for the Japanese population. We, therefore, assume that other factors, such as variation in local parasitism pressure and parasitism risk (Davies & Brooke, 1989b; Lindholm & Thomas, 2000; Stokke et al., 2008; Thorogood & Davies, 2013; Welbergen & Davies, 2012; Yang et al., 2015), host individual experience (Campobello & Sealy, 2011; Capek et al., 2010) and others, could better explain observed differences. In the great reed warbler population in Slovakia, the parasitism rate averaged 35.4% (Trnka, Požgayová, et al., 2012; Trnka et al., 2013; Trnka & Grim, 2014, this study), which appears to be higher than parasitism rates found in the studied populations of the Oriental reed warbler in China and the overall rate of cuckoo parasitism estimated from local parasitism rates across various geographic populations of this cuckoo host in China (23.7%, Li et al., 2015; Li, Ruan, et al., 2016; Li, Zhang, et al., 2016; Li et al., 2020; Ma, Yang, Liu, et al., 2018; Yang et al., 2014, 2017). Furthermore, host defenses against cuckoo parasitism may also be associated with spatiotemporal variation in parasitism risk through host phenotypic plasticity (Campobello & Sealy, 2010). Indeed, while local variation in parasitism risk resulting from varying distances of particular host nests from potential cuckoo perches seems to be negligible in the study site in Slovakia, which consists of narrow belts of littoral vegetation lined with trees running parallel and at an equal distance from the reeds (Trnka & Grim, 2014), nests of Oriental reed warblers at study sites in China were located at different distances from cuckoo perches and thus, they may be exposed to different risks from cuckoo parasitism.

Age and previous experience of hosts with the common cuckoo parasitism may be an additional factor influencing host antiparasitic defenses. Generally, the intensity of host defenses is expected to be higher in older and more experienced hosts compared with the younger birds because of lower future reproductive potential of older individuals and their cumulative experience due to possible former interactions with the cuckoo (Capek et al., 2010; Lotem et al., 1992; Molina‐Morales et al., 2014; Moskát et al., 2014; but see e.g., Procházka et al., 2014). Although there is no evidence for the effects of host age and host individual experience on egg discrimination and aggression against adult cuckoos in the studied great reed warbler population (Procházka et al., 2014; Trnka et al., 2013), further studies focusing specifically on these potential factors in the Oriental reed warbler would be valuable. Whether other factors are responsible for obtained findings remains to be investigated.

Hence, we can conclude that both the great reed warbler and Oriental reed warbler have well‐developed antiparasitic behaviors against common cuckoos in the studied populations, but with different levels of defense intensity, which may be due to local differences in parasitic pressure and the risk of parasitism. Moreover, other ecological factors and population attributes such as habitat heterogeneity, host density, and immigration (Moskát, Hansson, et al., 2008), may have contributed to these differences. Thus, despite the probably different coevolutionary history of the two *Acrocephalus* warblers with the common cuckoo, we assume that these two host‐brood parasite systems are at the same stage in the arms race, at least as far as the central European and northeastern Chinese populations are concerned. This provides a good opportunity to study coevolutionary processes between the common cuckoo and its hosts together in both species at large geographical scales and allows us to gain deeper insight into the problem of coevolution as such. Nevertheless, more studies with larger sample sizes and on more populations of the great reed warbler and Oriental reed warbler in various environmental conditions are required before a general conclusion can be drawn.

## AUTHOR CONTRIBUTIONS

**Alfréd Trnka:** Conceptualization (equal); formal analysis (equal); investigation (equal); writing – original draft (lead); writing – review and editing (equal). **Laikun Ma:** Formal analysis (equal); investigation (equal). **Hanlin Yan:** Investigation (equal). **Longwu Wang:** Investigation (equal). **Wei Liang:** Conceptualization (equal); funding acquisition (lead); resources (equal); writing – review and editing (equal).

## FUNDING INFORMATION

This work was supported by the National Natural Science Foundation of China (Nos. 32101242 to LM, 31960105 and 32260253 to LW, 31970427 and 32270526 to WL). LW was funded by the Guizhou Natural Science Foundation (No. ZK [2022]‐316), and WL was supported by the specific research fund of The Innovation Platform for Academicians of Hainan Province.

## CONFLICT OF INTEREST STATEMENT

The authors declare that they have no competing interests.

## ETHICS STATEMENT

The study was conducted in compliance with the law of Slovakia and China. The licenses and permissions to handle and ring the birds in Slovakia were issued by the Ministry of Environment of the Slovak Republic (number 3320/2019–6.3). Experimental procedures in China were in accordance with the Animal Research Ethics Committee of Hainan Provincial Education Centre for Ecology and Environment, Hainan Normal University (no. HNECEE‐2012‐003) and Guizhou Normal University (No. GZNUECEE‐2021‐001).

## Data Availability

Data and 3D files used for this manuscript are available at the Dryad Digital Repository: https://datadryad.org/stash/share/vK5nML5v6S2YIVYkpiKP40H9J6DGob3aMtAl8GUGV1Q.
